# Reproductive cycles and reproductive strategies among populations of the Rose‐bellied Lizard *Sceloporus variabilis* (Squamata: Phrynosomatidae) from central Mexico

**DOI:** 10.1002/ece3.1998

**Published:** 2016-02-18

**Authors:** Raciel Cruz‐Elizalde, Aurelio Ramírez‐Bautista

**Affiliations:** ^1^ Centro de Investigaciones BiológicasInstituto de Ciencias Básicas e IngenieríaUniversidad Autónoma del Estado de HidalgoCarretera Pachuca‐Tulancingo Km 4.5, Colonia CarbonerasC. P. 42184Mineral de La ReformaHidalgoMéxico

**Keywords:** Geographic variation, life history, lizard, populations, reproduction

## Abstract

Species with wide distribution, generally show variations in life history characteristics, which can be attributed to environmental causes. In this study, we analyzed the reproductive cycle and reproductive characteristics from three populations (Atlapexco, San Pablo Tetlapayac, and Santa Catarina) of the lizard *Sceloporus variabilis* from central Mexico. The specific goal of this study was to evaluate life history characteristics such as reproductive period extent, SVL (snout‐vent length) at sexual maturity, clutch size, egg mass and volume, and RCM (relative clutch mass). The San Pablo Tetlapayac population showed a larger clutch size, RCM, egg mass, and a smaller SVL, body mass and reproductive period (January‐September), as well as egg volume than the Atlapexco and Santa Catarina populations. Reproductive cycle and reproductive characteristics were more similar between the Atlapexco and Santa Catarina populations. Differences found in the population of San Pablo Tetlapayac with respect to the Atlapexco and Santa Catarina populations could be attributed to environmental variations where lizard populations occur. Differences in the reproductive period and reproductive characteristics in each population could be the result of both historical (phylogenetic; e.g., reproductive mode) and nonhistorical (environmental; e.g., temperature, food availability) causes. This study showed that populations of the same species are under different selection pressures, and these affect the reproductive characteristics of populations. Our results also indicate that long‐term and targeted studies on predation, use and selection of food, are needed to determine the causes of these variations in populations of *S. variabilis*.

## Introduction

Variation in reproductive patterns, such as length of reproductive period, SVL (snout‐vent length) at sexual maturity, clutch size, and offspring SVL at birth in diverse lizard species has been well‐documented in the last decades (Ramírez‐Bautista and Vitt 1997; Hernández‐Salinas and Ramírez‐Bautista 2015; Lozano et al. 2014; Hernández‐Salinas and Ramírez‐Bautista 2015; Roitberg et al. 2015). These variations occur within (Ballinger 1977, 1979; Ferguson et al. 1980; Ramírez‐Bautista and Vitt 1997; Znari et al. 2002; Ramírez‐Bautista et al. 2015) and among populations widely distributed (Dunham 1982; Du et al. 2005, 2014; Wang et al. 2011; Horváthová et al. 2013; Hernández‐Salinas and Ramírez‐Bautista 2015; Hosseinian Yousefkhani et al. 2014; Hernández‐Salinas and Ramírez‐Bautista 2015; Roitberg et al. 2015) of a single species. Most of these studies were based on the pioneer work by Tinkle (1969) and Tinkle et al. (1970), who identified two groups of basic predictions about life history study, (1) small‐bodied‐sized species with short life, rapid growth rate, small clutch size, multiple clutches within a season, oviparity, smaller SVL at birth, and an association with tropical environments, versus (2) large‐bodied‐sized species with long life, slow growth rate, single clutch during the reproductive season, hatchlings with small/larger size at birth, oviparity or viviparity and an association with temperate or tropical environments.

The predictions of Tinkle (1969) and Tinkle et al. (1970) gave rise to studies comparing populations of species with wide distributions and there have been several studies to understand life history evolution (Horváthová et al. 2013; Hernández‐Salinas and Ramírez‐Bautista 2015; Roitberg et al. 2015). These studies focused on variations in life history traits such as length of the reproductive season, SVL and age at sexual maturity, growth rate, fecundity, survivorship, clutch size, and offspring SVL at birth (Tinkle 1969; Tinkle et al. 1970; Stearns 1992). Causes and consequences of life history variation among populations has been a central topic in evolutionary ecology for decades (Wang et al. 2011; Horváthová et al. 2013; Roitberg et al. 2015). Variations in life history traits have been attributed to several factors, such as food availability (Ballinger 1977; Naya et al. 2007), seasonality of rainfall (Blois et al. 2008; Marquiz et al. 2008), population density, and predation intensity (Jenkins et al. 1999; Hernández‐Salinas et al. 2014). These factors are believed to promote life history variation (Stearns 1992; Hosseinian Yousefkhani et al. 2014) among populations, the plasticity of these traits has been demonstrated for a variety of lizard species from tropical (*Sceloporus variabilis*, Benabib 1994; *Anolis carolinensis*, Michaud and Echternacht 1995; *A. nebulosus*, Hernández‐Salinas and Ramírez‐Bautista 2015) and temperate environments (*S*. *undulatus*, Du et al. 2014; *Phrynocephalus przewalskii*, Wang et al. 2011; *Zootaca vivipara*, Horváthová et al. 2013; *Lacerta agilis*, Roitberg et al. 2015).

Reproductive strategies, such as extension of reproductive cycles between males and females are linked according to their evolved reproductive mode, continuous or seasonal (Lozano 2013). Seasonal reproductive cycle is typical in lizard species from high latitude temperate environments (Gadsden and Estrada‐Rodríguez 2008) and high elevations (Guillette 1981, 1982; Rodríguez‐Romero et al. 2004) in which both oviparous and viviparous species occur (Guillette 1981). Some viviparous lizard species from high elevations reproduce in the fall, and males and females have asynchronous reproductive cycles (Guillette 1981; see Guillette et al. 1980). Many low elevation tropical and subtropical species have more or less continuous reproductive cycles, also with males and females synchronized in the reproductive activity (Benabib 1994; Ramírez‐Bautista et al. 2006). This pattern typically occurs in oviparous species, such as *Phyllodactylus lanei* (Ramírez‐Sandoval et al. 2006) and *Sceloporus variabilis* (Benabib 1994; Ramírez‐Bautista et al. 2006).

Lately studies on several lizard species, such as *Zootaca vivipara* (Roitberg et al. 2013) and species of the genus *Takydromus* (Du et al. 2005), *Phrynocephalus* (Wu et al. 2015), *Anolis* (Hernández‐Salinas and Ramírez‐Bautista 2015), and *Sceloporus* (Ouifero et al. 2007; Du et al. 2014; Lozano et al. 2015) have found variation on several reproductive traits. In this sense, oviparous species which have wide distribution are good models to asses the changes on diverses life history characteristics, and mainly in the extension on reproductive cycles.

**Figure 1:**
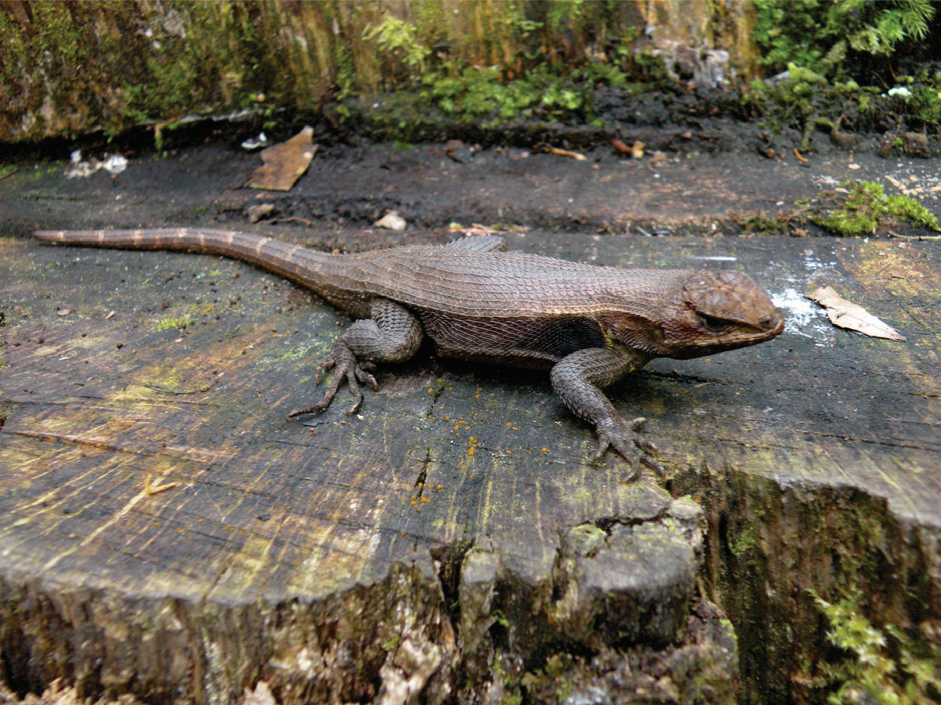
Male of *Sceloporus variabilis* from Hidalgo State, México.

In this study, we investigate life history traits among populations of the Rose‐bellied Lizard (*Sceloporus variabilis*; Fig. 1). This species is a small‐bodied‐sized lizard; on average, males have a SVL of 59.8 mm (range 48–71 mm) and females 51 mm (range 44–68 mm, Ramírez‐Bautista et al. 2006). This lizard has terrestrial habits, using tree trunks and rocks for perching. It has a broad geographic range distribution from Southern Texas, USA, through Mexico and Central America (Smith et al. 1993). In Mexico its distribution occurs from Yucatán, Oaxaca, Chiapas, Tabasco, Veracruz, Tamaulipas, Querétaro, San Luis Potosí and Hidalgo, at an elevation from sea level to 2000 m a.s.l. (Smith et al. 1993). Due to that inhabit contrasting environments, and because each population lives in a different environment, which represent various pressures on life history characteristics of species and populations of lizards (Horváthová et al. 2013; Du et al. 2014), we expect to find (1) differences in extension of reproductive period among populations, and (2) variation of reproductive characteristics (SVL of male and female at sexual maturity, clutch size, clutch frequency, egg volume, and relative clutch mass) among populations.

## Materials and Methods

### Study area

**Figure 2:**
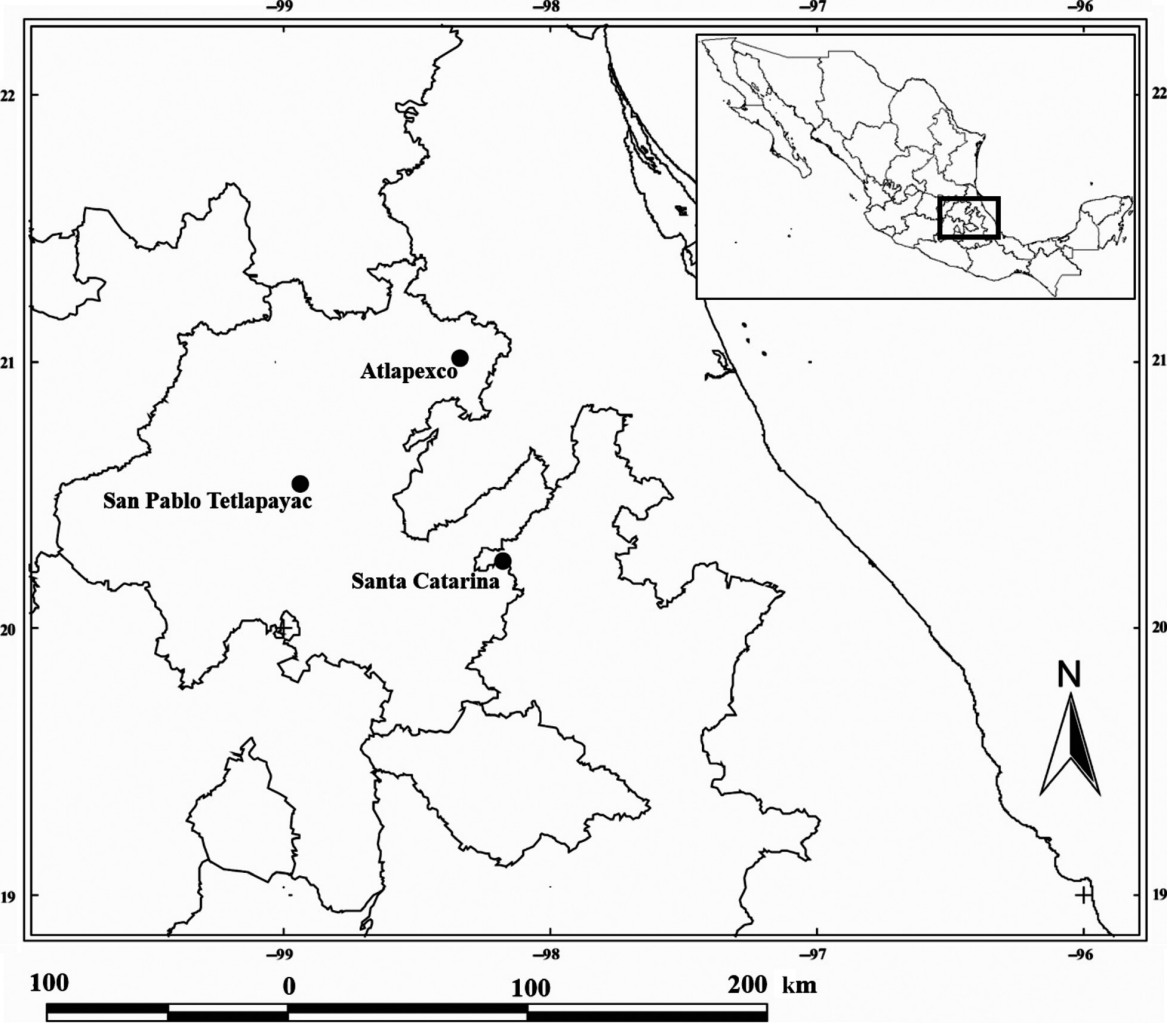
Map showing the localities of populations (Atlapexco, San Pablo Tetlapayac and Santa Catarina) included in this study, in central Mexico (figure on the top right inset).

This study was carried out in three geographical areas of Hidalgo State, Mexico: Atlapexco (98° 19′ 04′′N, 21° 09′ 45′′W, at an elevation of 140 m a.s.l.), San Pablo Tetlapayac (98° 55′ 14′′N, 20° 38′ 24′′W, 1045 m a.s.l.), and Santa Catarina (98° 11′ 31′′N, 20° 15′ 36′′W, 1845 m a.s.l.), located in the municipalities of Atlapexco, Metztitlán, and Acaxochitlán respectively. These localities differ in temperature, vegetation type, elevation, and precipitation (Table 1). The three populations are separated by a minimum straight‐line distance of 84.91 km (from Atlapexco to San Pablo Tetlapayac) and a maximum of 99.11 km (of San Pablo Tetlapayac to Santa Catarina; Fig. 2).

**Table 1:** Environmental characteristics from three localities of Hidalgo State, Mexico

Characteristics	Populations		
	Atlapexco	San Pablo Tetlapayac	Santa Catarina
Coordinates	98° 19′ 04′′N, 21° 09′ 45′′W	98° 55′ 14′′N, 20° 38′ 24′′W	98° 11′ 31′′N, 20° 15′ 36′′W
Elevation	140	1045	1845
Vegetation type	Tropical evergreen forest	Xeric scrublands	Cloud forest
Average annual precipitation (mm)	1500	700	608.5
Average annual temperature (°C)	22	18.5	14.5

### Field work

Sampling activities were conducted during each month (at the same time) for an entire year, from September 2013 to August 2014. Total sample size was 284 specimens, of which 277 were adults (defined in males if they had enlarged testes and convoluted epididymides consistent with sperm production, and presence of vitellogenic follicles in ovary, or eggs in oviducts in females; Goldberg and Lowe 1966; Ramírez‐Bautista et al. 2002) as follows: Atlapexco—110 lizards (55 males, 55 females), San Pablo Tetlapayac—79 lizards (42 males, 37 females), and Santa Catarina—88 lizards (40 males and 48 females). The specimens were differentiated by sex, males have postanal scales and ventral patches developed characteristic of various species of the genus *Sceloporus* (Ramírez‐Bautista et al. 2006). Specimens were collected under a scientific collecting permit issued by Secretaría del Medio Ambiente y Recursos Naturales (SEMARNAT; #SGPA/DGVS/11746/13). Lizards were euthanized in the laboratory by intracoelomic injection of sodium pentobarbital to prevent pain to individuals (this study was conducted according to the ethics and regulations for animal research of the Universidad Autónoma del Estado de Hidalgo and AVMA Guidelines on Euthanasia, 2013). Specimens were fixed in 10% formalin (Ramírez‐Bautista et al. 2008) and kept in the Laboratorio de Ecologia de Poblaciones of the Universidad Autónoma del Estado de Hidalgo.

### Morphological analysis

Morphological descriptions (SVL) and comparisons were limited to sexually mature males and females. We measured the snout‐vent length (SVL; to the nearest 1.0 mm) with a digital caliper (to nearest 0.01 mm) and body mass (g) with a pesola (to nearest 0.01 g) of males and females. Previous to analysis, data of SVL and body mass were transformed log_10_ to normalize the data and to eliminate the effect of SVL on body mass of lizards (Zar 1999; Schulte‐Hostedde et al. 2005). We used ANOVAs (Zar 1999) to test for sexual dimorphism in SVL and body mass between males and females from the three populations. Tukey's test was used to determine statistical difference in the mean value of SVL among populations.

### Reproductive analyses

For all adult specimens we removed gonads (testes in males, and nonvitellogenic follicles [previtellogenic follicles, NVF], vitellogenic follicles [VF] in ovary, and eggs [in oviduct] in females). The length and width of the gonads were used to calculate testicular and follicular volume (V), using the formula for the volume of an ellipsoid: V = 4/3*πa* ^2^ *b*, where *a* is one‐half the shortest diameter and *b* is one‐half the longest diameter (Ramírez‐Bautista et al. 2006). Testicular and follicular volumes were used as indicators of reproductive activity of males and females, similar to others studies (Ramírez‐Bautista et al. 2002, 2015). In addition, we removed and weighed (to nearest 0.0001 g) fat bodies and liver for both sexes to determine whether fat body and liver size fluctuates with changes in gonadal activity. In reproductive females, the largest ovarian follicles (NVF or VF) and eggs in the oviduct on both sides of the body, were weighed and multiplied by the number of follicles or eggs on that side to estimate the total gonad mass/volume or egg mass/volume (Ramírez‐Bautista et al. 2015). These values were used to estimate seasonal investment in reproduction. Smallest females containing enlarged vitellogenic follicles or oviductal eggs were used as estimates of minimum SVL at sexual maturity (Ramírez‐Bautista et al. 2008; Hernández‐Salinas et al. 2010). Males were considered sexually mature if they contained enlarged testes and highly convoluted epididymides, which are typically associated with sperm production (Lozano 2013).

To evaluate body‐size effects on reproductive variables, we first calculated regressions of log‐transformed organ volume (gonad) and organ mass (liver and fat body) on log‐male and log‐female SVL. For regressions that were significant (indicating a body mass size effect), we calculated the residuals from the relationship of organ volume/mass to SVL to produce SVL‐adjusted variables (Schulte‐Hostedde et al. 2005) and to ensure normality and homogeneity of variance by Shapiro‐Wilks test (by ShaphiroZar 1999). We used these residuals to describe organ and/or reproductive cycles. This technique maintains variation that is due to extrinsic factors (e.g., season) while minimizing the compounding effect of individual variation in SVL. For regressions that were not significant (e.g., no body size effect), we used logs of gonad volume to describe reproductive and/or organ mass, such as liver and fat body cycles (Ramírez‐Bautista and Vitt 1997; Hernández‐Salinas et al. 2010; Lozano 2013). We performed ANOVAs on values with month as the factor to determine if significant variation existed.

Clutch size was quantified by counting eggs in the oviduct of adult females during the reproductive cycle (Benabib 1994; Ramírez‐Bautista et al. 2006, 2015). Females with oviductal eggs and vitellogenic follicles simultaneously were considered to produce at least two clutches during the reproductive season (Ramírez‐Bautista et al. 2006, 2015). Additional evidence suggesting production of two or more clutches was indicated by presence of three classes of follicles in the ovary: class I (NVF) measuring 0.5–0.9 mm, class II (NVF) measuring 1.0–2.00 mm, and class III (VF) measuring >3.0 mm, and/or eggs in oviduct (Benabib 1994). In the reproductive tract of females, presence of corpora lutea and elongated and expanded oviducts indicated that eggs had been recently deposited (Benabib 1994). We calculated a Pearson′s correlation coefficient to test for a possible relationship between clutch size and the SVL of females (Zar 1999; Du et al. 2005). RCM (Relative clutch mass) was assessed by the formula RCM = *clutch mass/(body mass–clutch mass)* (Vitt and Congdon 1978). We used a significance value of *P *<* *0.05 for all analyses. Results are expressed as untransformed means ± SE. Statistical analyses were performed using StatView IV (Abacus Concepts 1992) and STATISICA 7.0 (StatSoft, Inc. Tulsa, OK). Tukey's test was used to determine statistical difference in the mean value of reproductive traits among populations.

## Results

### Snout‐vent length at sexual maturity

Males and females of *S. variabilis* in the three populations reached sexual maturity at different body size (Table 2). Minimum size at sexual maturity in males was lower at San Pablo Tetlapayac (42 mm) and Atlapexco (45 mm) than Santa Catarina (57 mm) populations, a similar pattern was recorded in females (Table 2). Mean body size in males was different among populations (*F*_2,131_ = 8.21, *P *<* *0.001; Table 3). Males from Santa Catarina were larger (SVL = 66.0 mm) than males from Atlapexco (SVL = 62.6 mm; Tukey's test, *P *=* *0.02) and San Pablo Tetlapayac (SVL = 60.2 mm; *P *<* *0.001) populations, and a similar pattern occurred in body mass (*P *<* *0.001; Table 2); in contrast, females were similar in SVL in the three populations (*F*_2,137_ = 2.45, *P *=* *0.089), but different in body mass (*F*_2,137_ = 4.68, *P *=* *0.010; Table 2) where only San Pablo Tetlapayac was different from Santa Catarina (Tukey's test, *P *<* *0.01).

**Table 2:** SVL (Snout‐vent length) and body mass of males and females from three populations of *Sceloporus variabilis. T*‐test, (significant, *P *<* *0.001, *nonsignificant)

Populations	*N*	SVL (mm)	*P*	Body mass (g)	*P*
Atlapexco					
Males	55	62.6 ± 0.93 (45–63)	<0.001	9.5 ± 0.44 (3–17)	<0.001
Females	55	53.57 ± 0.52 (45–63)		5.88 ± 0.18 (3–8.5)	
San Pablo Tetlapayac					
Males	42	60.2 ± 0.96 (42–68)	<0.001	8.26 ± 0.35 (2.5–11.5)	<0.001
Females	37	52.73 ± 0.51 (45–57)		5.34 ± 0.20 (3.2–8)	
Santa Catarina					
Males	40	66.0 ± 0.69 (57–73)	<0.001	11.29 ± 0.38 (6–15.5)	<0.001
Females	48	54.50 ± 0.57 (47–62)		6.22 ± 0.20 (4.5–11)	
Males SVL and body mass among populations		*F*_2,131_ = 8.21	<0.001	*F*_2,131_ = 10.96	<0.001
Females SVL and body mass among populations		*F*_2,137_ = 2.45	0.089*	*F*_2,137_ = 4.68	0.010

**Table 3:** Reproductive characteristics of females from three populations of *Sceloporus variabilis* in Hidalgo State, Mexico. ANOVA, * (<0.05), ** (<0.005), ns (nonsignificant)

Characteristics	Populations			
	Atlapexco	San Pablo Tetlapayac	Santa Catarina	*P*
Peak activity males	January–June, November–December	January–June, September	January–June, November– December	
Peak activity females	January–May, July–December	February–July, September	January–June, August	
Period of vitellogenic follicles	January–December	January–September	January–December	
Period of eggs production	January–November	February–September	January–June	
Mean number of VF	3.4 ± 0.18 (1–5, *n *=* *27)	4.4 ± 0.20 (3–6, *n *=* *18)	4.1 ± 0.21 (3–6, *n *=* *15)	*
Clutch size/Mean number of eggs	3.5 ± 0.19 (2–5, *n *=* *19)	4.3 ± 0.28 (3–6, *n *=* *12)	3.5 ± 0.24 (2–5, *n *=* *13)	*
Egg mass (g)	1.1 ± 0.09 (0.57–1.9, *n *=* *19)	1.4 ± 0.12 (0.88–2.1, *n *=* *12)	1.3 ± 0.13 (0.93–2.4, *n *=* *13)	ns
Egg volume (mm^3^)	698 ± 31.9 (387–904, *n *=* *19)	667 ± 41.3 (493–896, *n *=* *12)	751 ± 38.5 (552–988, *n *=* *13)	ns
RCM	0.207 ± 0.013 (0.131–0.328)	0.310 ± 0.019 (0.215–0.423)	0.254 ± 0.017 (0.183–0.390)	**

### Reproductive cycles

#### Males

**Figure 3:**
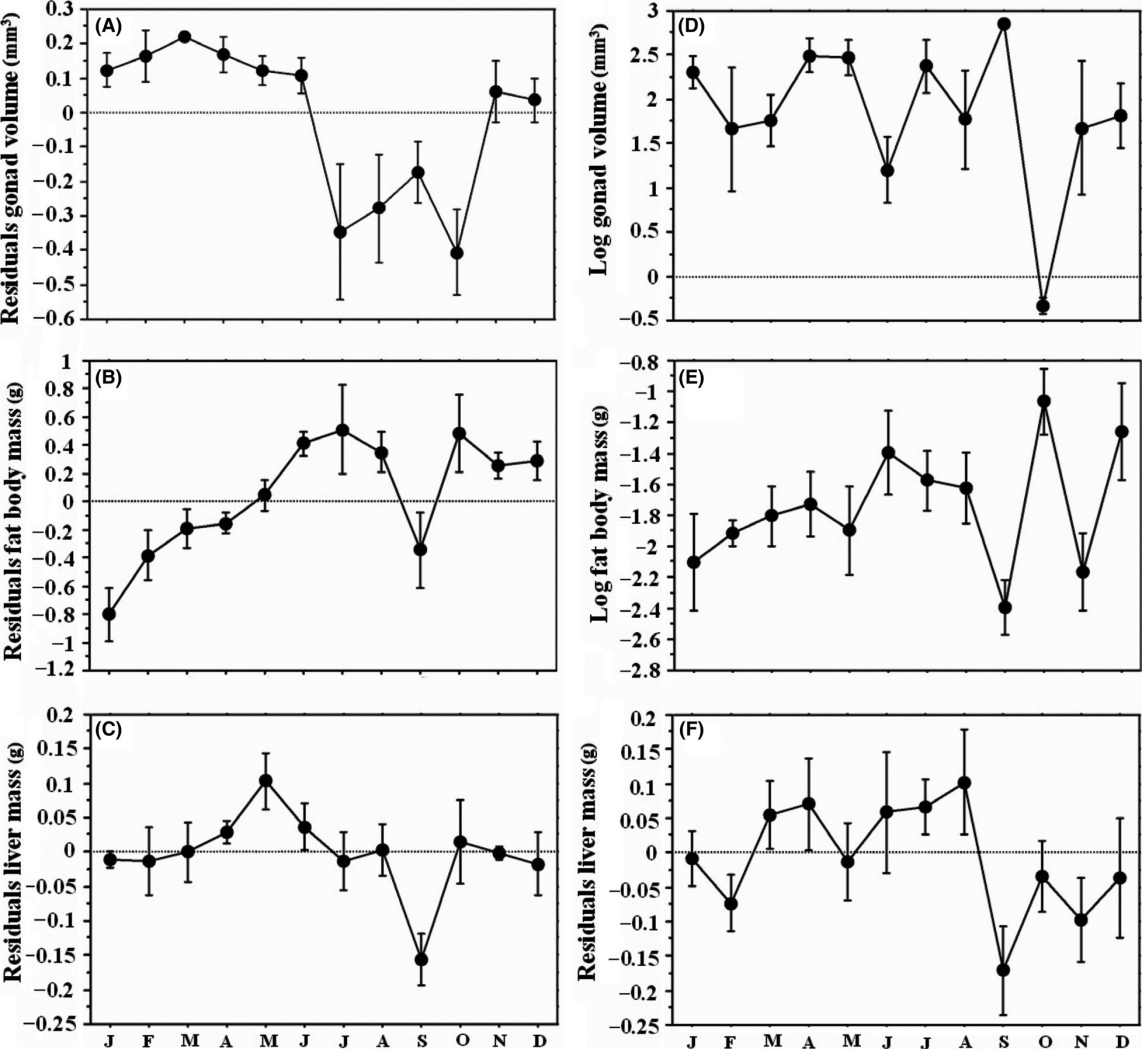
Monthly variation in volume of gonads, liver, and fat bodies in males (A–C) and females (D–F) of *Sceloporus variabilis* from Atlapexco, Hidalgo State, Mexico. Means given ± SE.

**Figure 4:**
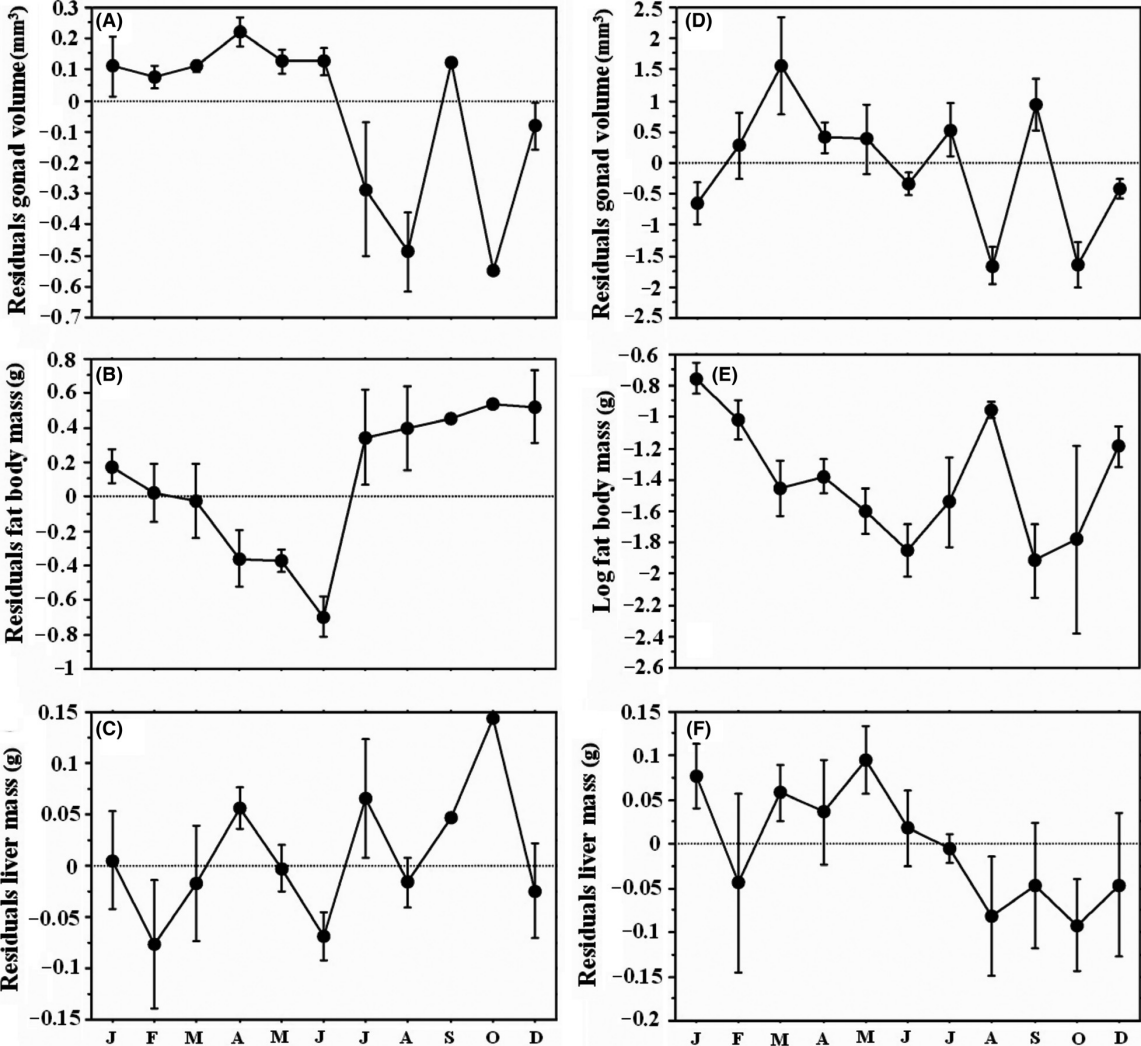
Monthly variation in volume of gonads, liver, and fat bodies in males (A–C) and females (D–F) of *Sceloporus variabilis* from San Pablo Tetlapayac, Hidalgo State, Mexico. Means given ± SE.

**Figure 5:**
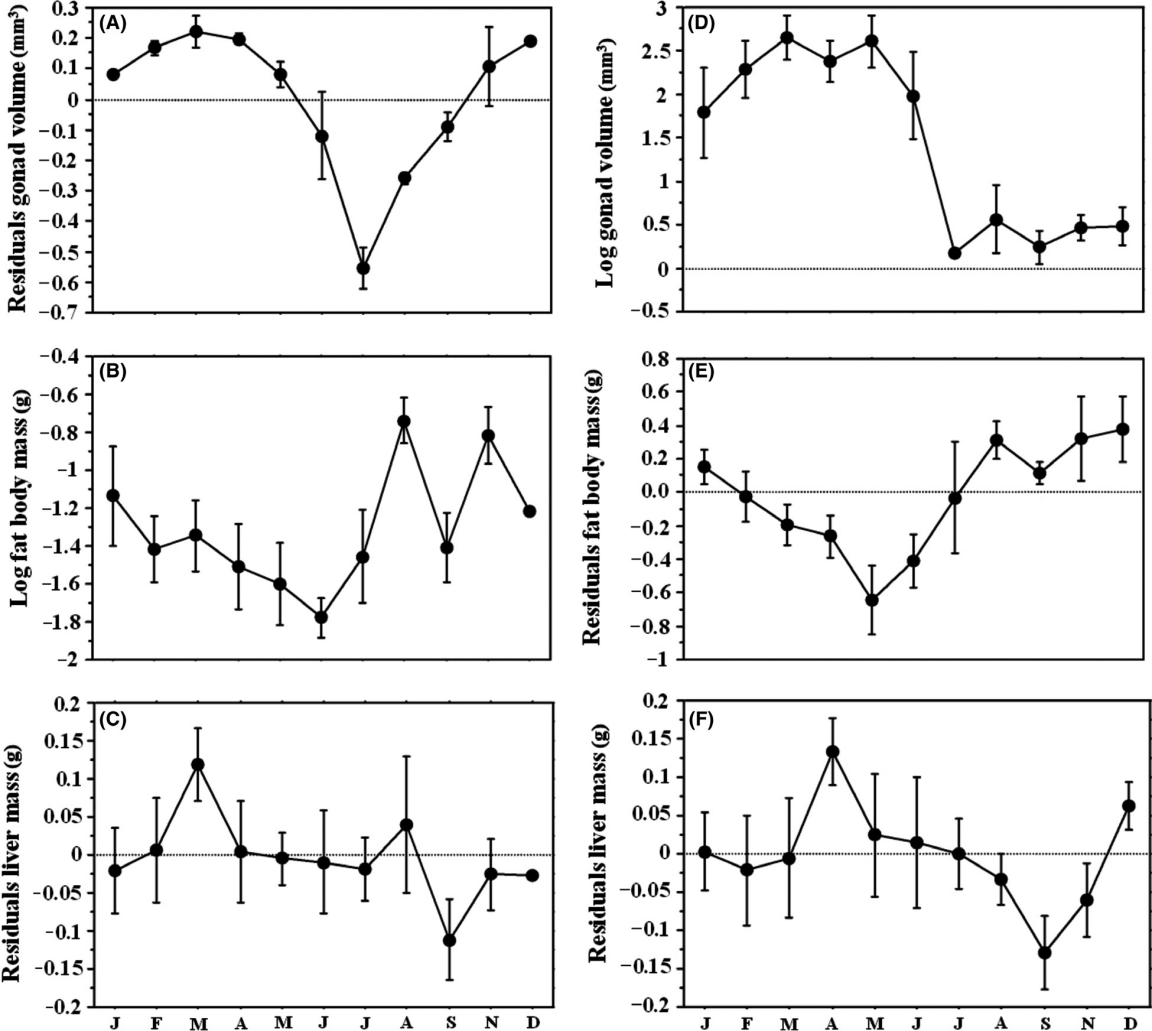
Monthly variation in volume of gonads, liver, and fat bodies in males (A–C) and females (D–F) of *Sceloporus variabilis* from Santa Catarina, Hidalgo State, Mexico. Means given ± SE.

A significant relationship existed between log_10_ SVL and log_10_ volume of testes (*r* ^2^ = 0.32, *F*_1_,_54_ = 24.5, *P *<* *0.001), log_10_ fat body mass (*r* ^2^ = 0.31, *F*_1,54_ = 23.5, *P *<* *0.001), and log_10_ liver mass (*r* ^2^ = 0.77, *F*_1,54_ = 176.9, *P *<* *0.001) in males from the Atlapexco population. Consequently, we used residuals of the regression to describe the testes, fat body mass, and liver mass cycles (Fig. 3). An ANOVA on residuals of the regressions revealed significant effects of month on volume testes (*F*_11,45_ = 5.73, *P *<* *0.001), fat body mass (*F*_11_,_43_ = 5.49, *P *<* *0.001), and liver mass (*F*_11_,_43_ = 2.21, *P *=* *0.031). Testes volume was higher from January to June, decreasing in July and August, but increasing again in September, with another peak in November and December (Fig. 3A). In males from San Pablo Tetlapayac, a significant relationship also existed between log_10_ SVL and log_10_ volume of testes (*r* ^2^ = 0.39, *F*_1,40_ = 25.58, *P *<* *0.001), log_10_ fat body mass (*r* ^2^ = 0.13, *F*_1,40_ = 5.76, *P *=* *0.021), and log_10_ liver mass (*r* ^2^ = 0.79, *F*_1,40_ = 145.9, *P *<* *0.001). An ANOVA on residuals of the regressions revealed significant effects of month on volume testes (*F*_10,31_ = 5.54, *P *<* *0.001), fat body mass (*F*_10,31_ = 4.52, *P *<* *0.001), but not liver mass (*F*_10,31_ = 1.48, *P *=* *0.194). Maximum testicular volume was observed from January to June, decreasing in July and August, and increasing again in September and December (Fig. 4A). Males from Santa Catarina followed a similar pattern. A significant relationship existed between log_10_ SVL and log_10_ volume of testes (*r* ^2^ = 0.11, *F*_1,38_ = 4.71, *P *=* *0.036), log_10_ liver mass (*r* ^2^ = 0.49, *F*_1,38_ = 36.18, *P *<* *0.0001), but not in log_10_ fat body mass (*r* ^2^ = 0.05, *F*_1,38_ = 1.97, *P *=* *0.168). An ANOVA on residuals of the regressions revealed significant effects of month on volume testes (*F*_10,29_ = 11.9, *P *<* *0.001), but not in fat body mass (*F*_10,29_ = 2.09, *P *=* *0.061), or liver mass (*F*_10,29_ = 0.796, *P *=* *0.633). The reproductive pattern of males from this population was well‐marked with two maximum peaks, one from January to May followed by a decrease in June and July, and a second increase during August. Maximum testes volume occurred in November and December (Fig. 5A).

Reproductive investment of males from the three populations is consistent in variations of fat body mass and liver mass throughout the year (Figs. 3, 4, 5). For example, when peak volume of testes of males from Atlapexco was reached early in the reproductive season, fat body mass was at its lowest (Fig. 3B), but began to increase as the reproductive season progressed; however, liver mass was maintained almost unchanged (Fig. 3C). Fat body mass of males from San Pablo Tetlapayac consistently decreased during the extended reproductive season (January–June), increasing again from July to December (Fig. 4B), when reproductive activity of males decreased. In contrast with males from Atlapexco, liver mass varied during the year (Fig. 4C). Finally, fat body mass of the males from Santa Catarina continually declined during the reproductive season reaching minimum size at the end of the reproductive season. This was followed by a concordant increase in both testes volume and fat body mass after the reproductive season (August–December; Fig. 5A and B). Liver mass varied little throughout the year (Fig. 5C).

#### Females

A significant relationship existed between log_10_ SVL and log_10_ liver mass (*r* ^2^ = 0.36, *F*_1,54_ = 29.5, *P *<* *0.001) for females from the Atlapexco population. No significant relationship was detected for log_10_ of gonad volume (*r* ^2^ = 0.054, *F*_1,54_ = 3.05, *P *=* *0.087) and log_10_ fat body mass (*r* ^2^ = 0.001, *F*_1,54_ = 0.041, *P *=* *0.839). As with males, we removed the effect of female size by using the residuals from the common regressions to describe the liver cycles, whereas the gonad volume and fat body mass cycles were best represented by log‐transformed values (Fig. 3D–F). The ANOVAs revealed significant effect of month on log_10_ gonad volume (*F*_11,43_ = 3.65, *P *=* *0.001), log_10_ fat body mass (*F*_11,43_ = 2.09, *P *=* *0.040), but not log_10_ liver mass (*F*_11,43_ = 1.69, *P *=* *0.107). Maximum reproductive activity of females was from January to September, during which females produced VF and eggs. Reproductive activity dropped abruptly in October, and increased again in November and December (Fig. 3D). In females from San Pablo Tetlapayac there was a significant relationship between log_10_ SVL and log_10_ volume of the gonad (*r* ^2^ = 0.35, *F*_1,40_ = 21.3, *P *<* *0.001) and log_10_ liver mass (*r* ^2^ = 0.55, *F*_1,40_ = 47.5, *P *<* *0.001), but not log_10_ fat body mass (*r* ^2^ = 0.02, *F*_1,40_ = 0.677, *P *=* *0.415). We removed the effect of female size by using the residuals from the common regressions to describe the gonad and liver mass cycles, whereas the fat body mass was best represented by log_10_ (Fig. 4D–F). The ANOVAs on residuals of the regressions revealed a significant effect of month on gonad volume (*F*_10,30_ = 4.82, *P *<* *0.001) and fat body mass (*F*_10,30_ = 4.65, *P *<* *0.001), but not liver mass (*F*_10,30_ = 0.687, *P *=* *0.728). During reproductive activity, maximum VF and eggs production by females was from January to September, decreasing in October and December (Fig. 4D). For females from Santa Catarina, there was a significant relationship between log_10_ SVL and log_10_ fat body mass (*r* ^2^ = 0.15, *F*_1,49_ = 8.7, *P *=* *0.005), log_10_ liver mass (*r* ^2^ = 0.61, *F*_1,49_ = 76.9, *P *<* *0.001), but not log_10_ volume of the gonad (*r* ^2^ = 0.06, *F*_1,49_ = 2.82, *P *=* *0.098). We removed the effect of female size by using the residuals of the regressions to describe the fat body and liver cycles, whereas the gonad was best represented by log‐transformed data (Fig. 5D–F). The ANOVAs on residuals of the regressions revealed a significant effect of month on gonad volume (*F*_10,40_ = 9.47, *P *<* *0.001), fat body mass (*F*_10,40_ = 3.33, *P *=* *0.003), but not liver mass (*F*_10,40_ = 1.42, *P *=* *0.210). Female reproductive activity began from January to June, when maximum VF and eggs production occurred; however, production of VF occurred throughout the year (Fig. 5D).

Fat body and liver mass remained relatively high during female reproductive activity in lizards from the Atlapexco population (Fig. 3E and F). Fat body mass was low during reproductive activity for females from San Pablo Tetlapayac (Fig. 4D and E) but liver mass fluctuated very little during maximum reproductive activity (Fig. 4F). Finally, fat body mass for females from Santa Catarina decreased with increasing reproductive activity (Fig. 5D and E), and increased when reproductive activity decreased (Fig. 5D). Liver mass was almost constant during reproductive activity, except in April (increased) and September (decreased; Fig. 5F).

### Vitellogenic follicles and eggs production

**Figure 6:**
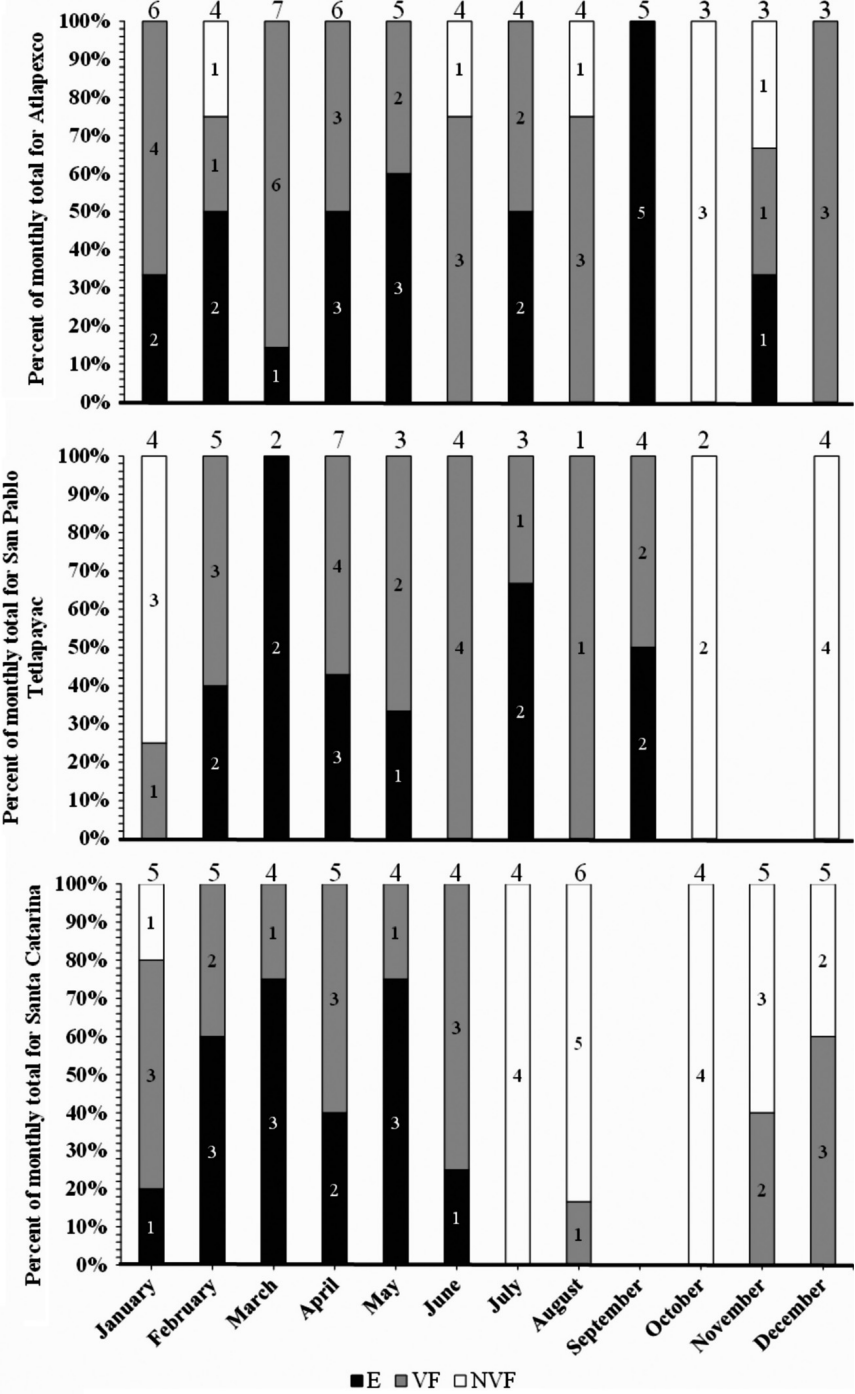
Seasonal changes in frequencies of various reproductive states for female of *Sceloporus variabilis* from Atlapexco, San Pablo Tetlapayac and Santa Catarina populations, Hidalgo State, Mexico. Sample size of each month is above the bars; numbers inside de bars are egg classes (E = eggs, VF = vitellogenic follicles, NVF = nonvitellogenic follicles).

Vitellogenic follicle and egg production by females varied among populations (Fig. 6; Table 3). Females from the Atlapexco population produced VF during January–December and eggs during January–November (Fig. 6); females from San Pablo Tetlapayac produced VF during January–September and eggs during February–September (Fig. 6; Table 3); finally, females from the Santa Catarina population produced VF during the entire year, but eggs were found only during January–June (Fig. 6, Table 3).

### Clutch size and frequency

Mean clutch size varied among populations (*F*_2,41_ = 4.48, *P *=* *0.017; Table 3). Clutch size by females from Atlapexco (3.5 ± 0.21, *n *=* *19) was similar to that of Santa Catarina females (3.4 ± 0.24, *n *=* *13; *P *=* *0.993), and both of these were lower than those of San Pablo Tetlapayac females (4.3 ± 0.28, *n *=* *12, Table 3) (Tukey's post hoc test, *P *=* *0.030 and *P *=* *0.023, respectively). Females with VF were similar in SVL (53.9 ± 0.43, *n *=* *66) to females with oviductal eggs (53.7 ± 0.53, *n *=* *44; Mann**–**Whitney *U*‐test, Z = −0.528, *P *=* *0.597). Clutch size was correlated with female SVL for the Atlapexco population (*r* ^2^ = 0.48, *F*_1,17_ = 15.46, *P < *0.001) but not for the San Pablo Tetlapayac (*r* ^2^ = 0.20, *F*_1,10_ = 2.49, *P *=* *0.145) or Santa Catarina (*r* ^2^ = 0.16, *F*_1,11_ = 2.15, *P *=* *0.171) populations.

Clutch frequencies varied among populations. For example, the Atlapexco and San Pablo Tetlapayac populations produced three clutches (NVF type II, VF in ovary and oviductal eggs), whereas the Santa Catarina population produced four clutches (NVF type I and II, vitellogenic follicles and oviductal eggs simultaneously). RCM varied among populations (ANOVA, *F*_2,41_ = 10.9, *P *<* *0.001; Table 3). RCM for the San Pablo Tetlapayac population was higher than that of the Atlapexco (Tukey's test, *P *<* *0.001) and Santa Catarina (*P *=* *0.04) populations (Table 3), and Atlapexco and Santa Catarina were similar (*P *=* *0.10). RCM also varied among months. During November, Atlapexco had the lowest RCM (0.145), whereas the highest RCM (0.310) was observed in July. Females from San Pablo Tetlapayac had the lowest RCM (0.262) in May and the highest (0.361) in July. Females from Santa Catarina had the lowest RCM (0.183) in June and the highest (0.300) in February. Egg volume (mm^3^) was not different among populations (*F*_2,41_ = 1.14, *P *=* *0.328). However, the lowest mean egg volume occurred in the San Pablo Tetlapayac females (Table 3), and the highest was in the Santa Catarina females (Table 3).

## Discussion

Theory suggests that species with broad distributions should express phenotypic variation in life history traits among populations determined by ecological and genetic factors (temperature, precipitation, food availability, or reproductive mode of species; Stearns 1992; Horváthová et al. 2013; Hosseinian Yousefkhani et al. 2014; Roitberg et al. 2015). Ecological factors influence local differentiation in morphological and reproductive traits among populations and are usually considered adaptive in nature (Du et al. 2005; Wang et al. 2011; Horváthová et al. 2013; Hosseinian Yousefkhani et al. 2014; Roitberg et al. 2015). Populations of *S. variabilis* from central Mexico (this study; 140, 1045 and 1845 m elev.) appear to be good models for examining variations in morphology and reproductive characteristics, and these differ from studied populations in eastern Mexico near the coast (Benabib 1994; Ramírez‐Bautista et al. 2006). Our study showed variation in morphological (SVL at sexual maturity, mean SVL, body mass) and reproductive characteristics (reproductive cycles, clutch size, clutch frequency, and RCM) among populations of *S. variabilis* analyzed, a similar pattern has been observed in other species of lizards (Du et al. 2005; Roitberg et al. 2015). These variations in reproductive traits may be a response to the environments (temperature, elevation or vegetation type; Table 1) where lizard populations inhabit, such as occur in other populations of the same species (Benabib 1994; Ramírez‐Bautista et al. 2006).

### Body size at sexual maturity

*Sceloporus variabilis* is a small‐body‐sized lizard. Males and females from the three populations reached minimum SVL at sexual maturity at different sizes. The smallest sexually mature females were from San Pablo Tetlapayac (45.3 mm) and were similar to those from Atlapexco (45.4 mm). The largest were from the Santa Catarina population (47.1 mm). Because of the correlation between SVL and fecundity (yearly reproductive output) across populations and the lack of differences among populations in egg size, differences in fecundity most likely reflect the impact of female body size on fecundity. Environments in which these populations live are considerably different. Santa Catarina is represented by a high elevation (1845 m) cloud forest with lower temperatures (14.5°C) and precipitation (608 mm) than Atlapexco with tropical evergreen forest at low elevation (140 m) with higher temperatures (22°C) and precipitation (1500 mm; Table 1). San Pablo Tetlapayac contains xeric scrubland, which is intermediate in temperature and precipitation relative to the other populations (Table 1). Each population responds differently in terms of reproductive characteristics to these different environmental conditions, and these can be affected by other factors such as foraging ability, food availability and habitat utilization, which has been documented in other lizard species and populations (Benabib 1994; Du et al. 2005, 2014; Wang et al. 2011; Horváthová et al. 2013; Hosseinian Yousefkhani et al. 2014; Roitberg et al. 2015). A similar pattern is evident in males from the three populations. Santa Catarina and Atlapexco males were larger in mean SVL and minimum size at sexual maturity than males from San Pablo Tetlapayac.

In our study, size at sexual maturity could also result from the extension of the reproductive activity in each population as is observed in other lizard studies (Ramírez‐Bautista and Vitt 1997, 1998). Females from San Pablo Tetlapayac reach minimum SVL at a smaller size resulting in production of VF and eggs during the first month of the year, similar to the Atlapexco populations (see cycles; Figs. 3, 4). This reproductive pattern of sexual maturity at smaller sizes has been observed in other populations of this species in tropical environments (Benabib 1994; Ramírez‐Bautista et al. 2006) and in different oviparous species as *Anolis nebulosus* (Ramírez‐Bautista and Vitt 1997, 1998; Hernández‐Salinas and Ramírez‐Bautista 2015). For example, in two populations of *A. nebulosus* near the Pacific Coast, females from two populations showed different size at sexual maturity, explained by different pressure of depredation, or food availability. Rapid growth rate in order to reach minimum SVL to reproduce is common in oviparous lizards with small body size (Ramírez‐Bautista 1995). This allows greater clutch frequencies, suggesting a beat‐heading life‐history strategy (Stearns 1992), where males and females of the three populations can grow rapidly before the beginning of the reproductive season (Benabib 1994; Ramírez‐Bautista and Vitt 1997, 1998).

### Reproductive cycles

Males and females of *S. variabilis* had nearly continuous reproductive cycle during the year, similar to other populations from the Gulf coast of Mexico (García‐Collazo et al. 1993; Benabib 1994; Ramírez‐Bautista et al. 2006). However, reproductive cycles of males varied in extension among populations and also differed in the same species studied elsewhere (from February–May and October–December reported by Benabib 1994; Ramírez‐Bautista et al. 2006). Males from Atlapexco were reproductively active during most of the year with two peaks, from January to June and November–December (Fig. 3A). The reproductive cycle of males from San Pablo Tetlapayac differed slightly, with maximum testes volume occurring from January to June, and another slight peak in September (Fig. 4A). Males from Santa Catarina reproduced continually with two distinct peaks, one from January–June and the other from August to December (Fig. 5A). Variations in reproductive activity among populations likely are a response to temperature, precipitation, or food availability in the respective environments (Ballinger 1977; Dunham 1978; Ramírez‐Bautista 1995; Ramírez‐Bautista and Vitt 1997), or a combination of these factors (Licht and Gorman 1970; Ramírez‐Bautista and Vitt 1997).

Continuous reproduction by males of *S. variabilis* likely results in high energy cost as in other species (Ballinger 1977; Ramírez‐Bautista and Vitt 1997). This is supported by fat body mass but not liver mass cycles of males from the three populations (Figs. 3B and C, 4B and C, 5B and C). During reproduction, males invest a high amount of energy in copulation to the most number of females to ensure their fitness as well as the defense of territory and combats with other males (Ramírez‐Bautista et al. 2002; Stephenson and Ramírez‐Bautista 2012). In these populations of *S*. *variabilis*, fat body mass decreased (with slight changes) when reproductive activity was maximal, but liver mass remained nearly constant, except in males from San Pablo Tetlapayac, in which liver mass varied during the year (Fig. 4C). The increase of fat body and liver mass at the end of the reproductive cycles in the three populations, suggests that males not only are reproducing but also foraging, similar to other lizard species (Ramírez‐Bautista et al. 2006, 2014). A high energetic cost in reproduction has been shown in males and females from tropical oviparous species with continuous and synchronous reproduction between males and females; for example *Hemidactylus turcicus* (Selcer 1987, 1990), *S. pyrocephalus* (Ramírez‐Bautista and Olvera‐Becerril 2004), *P. lanei* (Ramírez‐Sandoval et al. 2006), and also in *S. variabilis* from the coast of Gulf Mexico (Benabib 1994; Ramírez‐Bautista et al. 2006). These cost are reflected in the number of events (clutches) that these species show in these environments, which also could be a reflect of the environment pressure (Hernández‐Salinas and Ramírez‐Bautista 2015). This pattern occurred in males and females of *S. variabilis* from the three studied populations according to cycles of liver mass and fat body mass in each population (Figs. 3, 4, 5).

Variation in reproductive cycles of females from the three populations was reflected in VF and eggs production (Fig. 6). Females from Atlapexco had two reproductive peaks, January–May and July–December, in which VF and eggs were produced (Fig. 6). Females from San Pablo Tetlapayac produced VF and eggs from February to September, whereas in Santa Catarina, females produced VF and eggs during a shorter time period (January–June). During the remaining months (July–December), these females had low production of VF. Even though we did not observe eggs in these females, we observed corpora lutea, which indicated that those females had ovulated (Fig. 6), also corpora lutea has been an indirect method to detect eggs production and clutch size in oviparous lizard species, and evidence of number of frequencies of clutches (Ramírez‐Bautista et al. 2006). Although continuous reproduction is typical in this species (Table 4), production of VF and eggs during the year varied among populations, similar to other populations (García‐Collazo et al. 1993; Benabib 1994; Ramírez‐Bautista et al. 2006; Table 4).

**Table 4:** Reproductive characteristics of female of *Sceloporus variabilis* from different populations including data from this study

SVL (mm)	Clutch size	Reproductive season	Resource
53.1 ± 0.49 (44–68)	4.6 ± 0.14 (3–7)	November–September	Benabib (1994)
55.4 ± 0.82 (53–61)	3.3 ± 0.9 (2–4)	–	Ramírez‐Bautista and González‐Romero (1991)
44–59	3.4 ± 0.57 (2–5)	January–December	García‐Collazo et al. (1993)
56.6 ± 0.35 (43.8–71)	3.7 ± 0.11 (2–6)	January–December	Ramírez‐Bautista et al. (2006)
53.57 ± 0.52 (45–63)	3.4 ± 0.21 (2–5)	January–December	Atlapexco, this study
52.73 ± 0.51 (45–57)	4.3 ± 0.28 (3–6)	January–September	San Pablo Tetlapayac, this study
54.50 ± 0.57 (47–62)	3.4 ± 0.24 (2–5)	January–December	Santa Catarina, this study

### Clutch size

Clutch size varied among these populations and varied relative to other populations of the same species as reported by Ramírez‐Bautista and González‐Romero (1991; 3.3 eggs), Benabib (1994; 4.3 and 4.6), Ramírez‐Bautista et al. (2006; 3.7), and García‐Collazo et al. (1993; 3.4). Mean clutch sizes in Atlapexco and Santa Catarina populations are similar to populations from the Gulf coast of Mexico, except from San Pablo Tetlapayac, which is similar to the population at Bastonal (Benabib 1994; 4.6 eggs; Table 4). Variation in clutch size within populations of coast and mainland suggests an environmental effect in clutch size among populations of *S. variabilis*; since differences in the population from San Pablo Tetlapayac were observed indicating this case. Moreover, this species has multiples clutches (3–4) during the year, and it is not well known how large each clutch is throughout the year (Benabib 1994). Similarities in clutch size between Atlapexco and Santa Catarina populations of this study and the differences in San Pablo Tetlapayac populations suggest that factors such as food availability, latitude and elevation (thus temperature) that these populations encounter could influence clutch size. Also, the medium elevation population (San Pablo Tetlapayac; 1045 m elev.) had similar mean clutch size compared with other high elevation populations (Bastonal; 1000 m elev.; Benabib 1994). Clutch frequency likely varies among populations in response to variation in environments where lizards live (Benabib 1994). Larger clutch size has been documented in other species of *Sceloporus* from high elevations, such as *S. jarrovii* (Ramírez‐Bautista et al. 2002), and *S. undulatus* (Angilletta et al. 2004).

The low average number of eggs in the analyzed populations of *S. variabilis* compared with other species of the genus from low elevations, such as *S. siniferus* (5.0 eggs; Fitch 1978; Ramírez‐Bautista et al. 2015) and *S. pyrocephalus* (5.8; Ramírez‐Bautista and Olvera‐Becerril 2004) may be compensated for by production of several clutches during the year (at least two or three) as in San Pablo Tetlapayac and Santa Catarina. Lizards from each population that we studied appear to distribute reproductive effort in similar ways (assuming each clutch size is similar) in terms of clutch frequency. For example, no differences were found in eggs mass or volume among the three populations. RCM did vary among populations; San Pablo Tetlapayac had the highest values (0.310). This suggests that each population may experience different levels of risk when active or may differ slightly in foraging mode (Vitt and Congdon 1978; Benabib 1994). However, RCM typically varies little across a wide range of body sizes within and among populations of single species (Vitt and Congdon 1978; Shine and Schwarzkopf 1992; Benabib 1994; Du et al. 2005; this study). This pattern may reflect a general tendency for female lizards, especially for small short‐lived species, which produce small clutch size, and multiples frequencies as *S. variabilis*. The significant differences in clutch size and RCM, and nondifferences in eggs mass among populations might be representing a trade‐off in these parameters, which also could be supporting the hypothesis that maternal body shape as well as size can be viewed as a response to fecundity (Vitt and Congdon 1978; Qualls and Andrews 1999; Du et al. 2005, 2014).

### In summary

Males and females from populations of *S. variabilis* are synchronized in production of mature gonads during the year. However, a relative shift in reproductive cycles of 1–2 months between males and females among these populations. The Atlapexco population differed from the San Pablo Tetlapayac and Santa Catarina populations. Energetic cost in reproduction of males and females is apparent in fat body and liver cycles which were at their minimum when reproduction was at its maximum, except in males and females from the Atlapexco population (Fig. 3). In this population, males and females may invest more time foraging while breeding as compared with other populations. Variation in liver mass is indicative of continuous foraging activity by the lizards, due to the liver synthesizing nutrients from fat bodies (Selcer 1987; Ramírez‐Bautista et al. 2004). Several factors might explain variation in clutch size and RCM among populations including temperature, precipitation, latitude, elevation, and food availability in the respective environments (Ballinger 1977). Females from high (Santa Catarina) and low (Atlapexco) elevation had larger SVL than females from medium elevation (San Pablo Tetlapayac), and also differences in life‐history characteristics were found (Table 3). These data suggest that females from these environments respond in different ways (extension of reproductive cycle, and variation in reproductive characteristics), as other lizard species do (Ramírez‐Bautista et al. 1995; Hernández‐Salinas and Ramírez‐Bautista 2015). On the other hand, variation in some of these characteristics (SVL, body mass, and reproductive characteristics) might reflect phenotypic plasticity among populations. Therefore, we suggest that lizards from each population respond to variation in predator pressure, food availability, temperature, or precipitation, also, these factors must be analyzed in these populations to determine whether changes are responses to the environment or adaptive responses of each population.

## Conflict of Interest

None declared.
